# Implementing Community Based Primary Healthcare for Older Adults with Complex Needs in Quebec, Ontario and New-Zealand: Describing Nine Cases

**DOI:** 10.5334/ijic.2506

**Published:** 2017-06-27

**Authors:** Mylaine Breton, Carolyn Steele Gray, Nicolette Sheridan, Jay Shaw, John Parsons, Paul Wankah, Timothy Kenealy, Ross Baker, Louise Belzile, Yves Couturier, Jean-Louis Denis, Walter P. Wodchis

**Affiliations:** 1 University of Sherbrooke, Canada; 2 University of Toronto, Canada; 3 University of Auckland, New Zealand; 4 École Nationale d’administration publique (ENAP), Canada

**Keywords:** primary healthcare, integrated care, older people, implementation, Québec, Ontario, New Zealand

## Abstract

The aim of this paper is to set the foundation for subsequent empirical studies of the “Implementing models of primary care for older adults with complex needs” project, by introducing and presenting a brief descriptive comparison of the nine case studies in Quebec, Ontario and New Zealand. Each case is described based on key dimensions of Rainbow model of Valentijn and al (2013) with a focus on “meso level” integration. Meso level integration is represented by organizational and professional elements of the Rainbow Model, which are of particular interest in our nine case studies. Each of the three cases in Ontario and three in New Zealand are different and described separately. In Quebec, a local health services network model is presented across the three cases studied with variations in the way it is implemented. The three cases selected in the three jurisdictions under study were not chosen to be representative of wider practice within each country, but rather represent interesting and unique models of community-based primary healthcare integration. Similarities and variations in the integrated care models, context and dimension of integration offer insights regarding core component of integration of services, offering a foundational understanding of the cases on which future analysis will be based.

## Introduction

Healthcare systems have turned to integrating their health and social services [1] in order to address specific problems of accessibility, continuity, quality and cost of services for people living with complex health, social and economic needs. Recent data suggest that population aging per se is not a major source of rising health cost, but rather that older adults disproportionately have complex care needs. Older adults with complex care needs often have multiple chronic illnesses and social needs, requiring access to a range of health and social services thereby increasing the complexity of organizing and coordinating care. Nearly half of adults aged 65 and over have more than one chronic condition [2, 3]. The presence of multiple chronic conditions is usually associated with worse health outcomes, greater utilization of healthcare services and higher healthcare costs [4]. Several empirical studies have demonstrated better patient satisfaction, continuity and quality of services in an integrated health system for older adults living with complex needs such as frailty, multiple chronic diseases, cognitive disorders and social isolation [5]. Moreover, similar studies demonstrated the efficiency and cost reduction of integrated health systems [6, 7]. Hence, a variety of models of integrated healthcare have been developed and piloted worldwide [8, 9, 11].

Contemporary literature reveals that the effective implementation of different models of integrated healthcare are influenced by factors such as the characteristics of the target population, existing and proposed processes and structures, which, in turn are influenced by organizational and policy environments [7, 10, 12]. The international research project *Implementing integrated care for older adults with complex health needs* (iCOACH), funded by grants from the Canadian Institutes of Health Research and the Health Research Council of New Zealand, aims at deepening the understanding of the steps to implementing innovative integrated community based primary healthcare models that address health and social needs, and improve outcomes for older adults with complex care needs, by carrying out three case studies in each of three different jurisdictions: Québec, Ontario and New Zealand. The three cases selected were not chosen to represent wider practices within each jurisdiction but because they offer insights into implementing models of integrated care in a range of contexts. The article by Kuluski and colleagues in this special issue reveals more information on the selection of cases [13]. The iCOACH project involves exploring case sites from multiple perspectives (policy makers, organizational managers, health and social care providers, patients and informal caregivers). The approaches to analyze cases are described in other articles within this issue (see Wodchis et al. for example) [14].

The primary aim of this paper is to set the foundation for subsequent empirical studies of the iCOACH project by introducing and presenting a brief descriptive comparison of the nine integrated care case studies for older persons with complex needs implemented in the three jurisdictions of this research project. The Rainbow Model of Integrated Care, developed to support systematic comparisons of integrated care models from a primary care perspective [15] was used to describe and compare components of the nine case studies. Graphic illustrations were used to portray the complex structures of each case. Although the focus of the broader program of work is on implementation of integrated models of care, this particular paper is focused on describing the cases based on the components of the Rainbow model to support analysis of implementation factors in future works.

## Conceptual framework

Integrating community-based primary healthcare services is an innovative way to organise and deliver health and social services in the primary care setting. Though the concept of “integration” has been thoroughly addressed by several studies [16, 17, 18], its operationalization has been more difficult. This may be due to “the complex nature of integrated care, which can never be fully rationalised nor standardised” [19]. The Rainbow Model of Integrated Care, the valuable work of Valentijn et al [15], was used as a descriptive framework for the cases studied in this project. This model distinguishes six interlinked integration dimensions: **clinical integration** (referring to clinical care coordination), **professional integration** (referring to inter-disciplinary coordination of services between various professionals), **organisational integration** (referring to the inter-organisational coordination of services between various organisations), **system integration** (referring to the alignment of rules and policies within a system), **functional integration** (referring to the coordination of support systems) and **normative integration** (referring to the extent of shared values and missions within the integrated system). The dimensions of integrated care presented in the model rest on the core principles of person-centred and population-focused care that underlies primary care delivery. Several constructs were recently operationalized in each dimension of the Rainbow Model of Integrated Care [19].

For the purposes of this descriptive comparison, we focus on “meso level” integration according to the Rainbow model, which is represented by organizational and provider level elements. System context is briefly discussed for each jurisdiction as this is a key to understanding how meso level integration is achieved. A more detailed analysis and description of system, or “macro level” integration is being offered in a separate paper discussing the policy context in each jurisdiction offer in this special issue. “Micro level” will be a point of emphasis in future works which should aim to describe the process and perception of clinical integration from the perspectives of providers and patients. Understanding how functional and normative processes support the macro, meso and micro levels of integration will be better addressed through an analytic perspective which will be achieved through subsequent empirical analyses of data collected through the case studies.

## Description of the three integrated case studies in Québec

### Quebec system context

Quebec’s jurisdiction has known many health and social system restructurations during last decades. To identify the substance of what follows, it is important to underline that the last reform in 2015, aiming to push further integrated care, was not taken into consideration to describe these case studies. The Québec government initiated a large-scale redesign of the organization of its healthcare structure in 2004, with the aim of implementing local health services networks across the province. The core of these local health services networks were 94 new organizations called Health and Social Services Centres (HSSC), that were created through the merger of several organizations operating within a defined geographical territory: Local Community Health Centers offering home care and social services, long-term care facilities and, in most cases, an acute care hospital [20]. These new organizations were formally mandated to lead the development of local health networks by encouraging the creation of formal and informal partnerships with various providers offering services to the population of their jurisdiction [21]. Local health networks were largely created through functional integration in the form of partnerships and alliances between autonomous organizations which have different levels of care, different missions and objectives such as community organizations, municipal organizations or private homecare organizations. HSSC have a great autonomy in terms of planning, organizing and managing services and activities [22] to meet the needs of the population served in their territory of jurisdiction [23]. Services are planned on individual and population based perspectives, structuring some services around the needs of select vulnerable populations such as older adults, mental health, persons with chronic illness cancer, etc. In fact, in Quebec, all Local community Health Centers head a local “integrated network for older adults” that spans its territory. All healthcare services under the HSSC are funded by the provincial health system but variable co-payments may apply for complementary services in the community and for long-term care facilities.

### Organizational Integration

The three cases studied in Québec are embedded in the same policy and system environment where a more top down strategy involving the creation of local health services networks was mandated. Each Health and Social Services Centre generally consists, as mentioned above, of several public establishments which were merged and brought under a single governance structure. For example, the highly urban local health network represented in one case has to interact with 30 establishments under the governance of HSSC such as hospital, several long-term care facilities and local community health centers with around 4700 employees compared to the rural local health network with 9 establishments and 1200 employees under de the governance of HSSC.

HSSC offers professional services in nursing, social work, rehabilitation, nutrition, among others, and some non-professional services like personal hygiene. Other services like meal preparation, meals-on-wheels, housekeeping and transportation are provided by private or community resources. Mostly, services are delivered in older adult’s homes.

**Figure 1:**
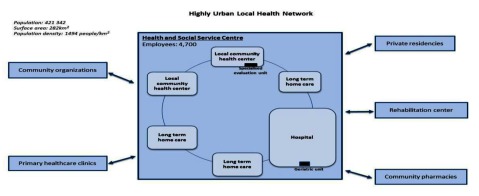
Highly urban Local Health Network.

**Figure 2:**
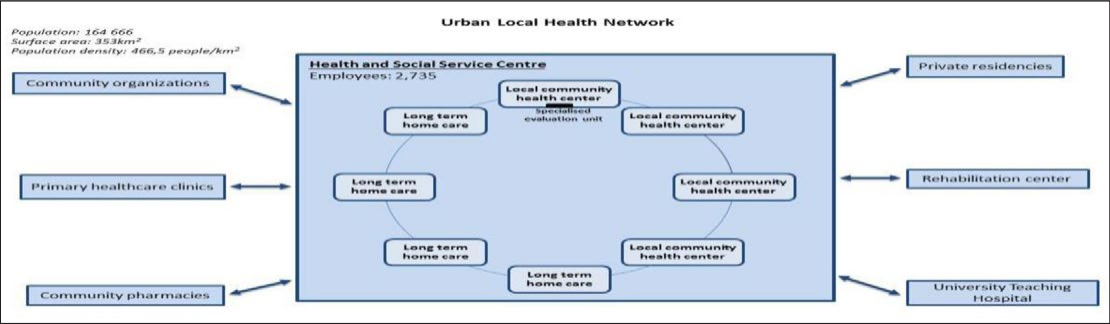
Urban Local Health Network.

**Figure 3:**
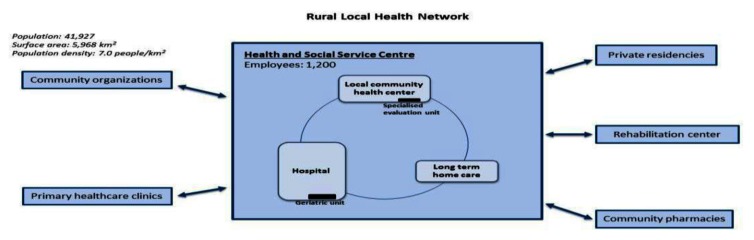
Rural Local Health Network.

Despite numerous similarities, many dimensions have to be considered to illustrate particular realities and challenges to achieve integration of services in each organisation. For example, according to territorial and population characteristics, the case under study will vary in terms of number and size of organization. This has an impact on the capacity of the organization’s leadership to create a common vision and a clear policy to promote collaboration. These three cases have the same overall model of integrated care, but structural differences are apparent as illustrated in Figures 1, 2 and 3.

The population served by the local health networks vary from more than 420 000 persons in the highly urban setting to around 40 000 persons in the rural setting. The challenge of the provision of services is not only based on the numbers of establishments involved and the number of persons but also in terms of the geographical characteristics of the organization. For instance, variable distances have to be covered to deliver care. The rural case covers a surface area of around 6000 km^2^ compared to the highly urban case with a surface area of around 280 km^2^. The various establishments involved in the local health network and the needs of the population vary also from one setting to another.

Vertical and horizontal governance is facilitated by a single high level manager who is responsible for addressing needs of a specific population through connecting a continuum of services. For example, this manager is responsible for older adults receiving care from home to long-term care facilities, including hospitalisation discharges. Another mechanism facilitating the development of inter organizational collaboration among the various organizations providing services in the local health networks is the implementation of a joint governing board under the leadership of each HSSC. Participating partners in this governing board include community organizations which deliver complementary assistance services such as housekeeping, transportation and caregiver support, owners of residential services in the community, community pharmacies, rehabilitation centers and family medicine practices of their territories. The joint governing board discusses and develops local projects in response to the needs of their population. This component illustrates that coordination is required at a high (governance) level, not only at the clinical level.

To optimize management and coordination of the offered services by the local health network, centralized points of access have been developed everywhere with a great incentive from the Ministry of Health and Social Services. This component of the integrated model ensures that professionals, physicians, family members as well as patients may directly contact the centralized point of access of their HSSC to obtain the right information they need about the public organization services.

### Professional integration

Most services in the continuum of care for older adults are provided by the HSSC. The majority of the services for older adults are offered by the home care and long-term care facilities subprogram. There are also health and social care providers (nurses, social workers, physiotherapists and occupational therapists) who work in the department of older adults affairs of the HSSC. Family physicians are also important primary healthcare providers in all the three cases in Quebec. However, most family physicians work in private clinics, and as such they are at the periphery of local health networks for older adults. In the three case studies, family physicians did not systematically participate in the local health network for older adults. In fact, the only family physicians involved in the local health network for older adults are those whose practice profile includes a substantial number of older adult with complex needs as clients or those practicing in Local community Health Center, long-term care facilities or day centers of their territories. In the rural case, it is a common practice for family physicians to do home care visits. Also, most patients in the rural case have been with their family physician for a long time. These long-lasting patient-physician relationships are less frequent in other highly-urban and urban cases.

The implementation of a common standardized comprehensive assessment tool, Multiclientele Assessment Tool, was supported by the Ministry of Health and Social Services (since 2000) and is a top priority in all three cases. This tool permits the evaluation of the functional autonomy of patients, and is not specific to any profession. Thus, it can be used by different professionals offering services to older adults with multiples needs in different organizations of the local health network. This shared clinical tool can facilitate interdisciplinary work by creating a common language (describing the needs of older people with complex needs) between professionals working in various organizations. Depending of the needs of the patients, social workers, nurses, occupational therapists and physiotherapists regularly form inter-professional teams which assess the patient’s needs and plan services to put in place. These teams are coordinated by case managers or main providers working in the HSSC.

Case management is an important component of this model of integration, as it aims at ensuring continuity and coordination of services. Even though many variations are observed in the implementation of case management across the three cases studied, they generally have good results such as an increased quality of care and continuity of services. A case manager is a stable figure and dedicated professional who coordinates all services needed by people with multiple needs, across organizations (from home to hospital) over time. Case management was loosely prescribed by the Ministry of Health and social services that can explain variations between case studies. The urban site has worked for many years to define and frame the practice of coordination. In this particular local health network, case managers are professional like social workers, nurses or occupational therapists, which means this role is not reserved for a specific profession; while in the highly urban network this function is assigned exclusively to social workers. The access to case management is different from site to site.

The high urban local network put in place a special team to be more efficient in management of waiting list for services. A specialized team composed of a nurse, a social worker, a respiratory therapist, a physiotherapist and an occupational therapist specialized in evaluation and orientation have been implemented. The team members quickly do the first evaluation of these clients with the same clinical assessment tool presented above. They either put services in place to solve urgent problems or orientate people to the appropriate services when necessary. The specialized services on evaluation and orientation can refer the patient for homecare services (long-term home care services from a provider) or to another health facility (eg to the hospital if the clinical state of the client is poor). In some cases, the hospital can request for an evaluation for a patient who is being discharged. In any case the hospital request still passes through the centralized point of access of the Health and social services centers.

Information sharing systems within and between organisations are yet to be realized, with no real alignment of different information systems and no inter-operability. Lacking information systems slows down implementation of an uninterrupted continuum of care with fluidity in the navigation of the health network. Though there have been significant advances in the implementation of clinical information systems in different points of services, there is still much ground to be covered in terms of interoperability.

These afore mentioned differences between what is spread everywhere, what is original answer (local innovation) to problems and how the same components are implemented differently from one site to another can be explained by the existence of a margin of local management autonomy despite a top-down high governance setting. Conversely, we can observe an authoritarian system at high level of governance and a space to adapt and to implement some local innovations.

## Three case studies in Ontario

### Ontario system context

In Ontario, medically necessary hospital and physician healthcare services are publicly-funded in a single payer healthcare system, under the oversight of the Ministry of Health and Long-term care (MOHLTC). Hospitals, long-term care, community health centres and home and community care services (delivered through 14 Community Care Access Centres in Ontario that coordinate home care services [26]) are paid for and governed by contracts between providers and one of 14 regional “Local Health Integration Networks.” Physician services are negotiated and paid for centrally through the Ministry of Health and Long-term care, as are medications, though the latter only covered for people over the age of 65 and those with permanent disability. While hospital, primary care, specialist and home and community care services are all available across Ontario, there is no requirement for these services to be linked or integrated. One approach to integrate care in Ontario are the Health Links, established in 2012 and intended to integrate hospital, physician and community services for the top 5% of health system users [24]. Aside from the development of Health Links, there has been considerable variation in the nature of integrated care initiatives, and the Ontario government has not mandated specific structures that connect services, as is the case in Quebec. Thus, integrated care models in Ontario can vary considerably with regard to structure, funding model and delivery method. With Health Links in their early implementation phases, three case studies of integrated care initiative in Ontario focused on other, more established models.

## Case – Community care agency

### Organizational integration

**Figure 4:**
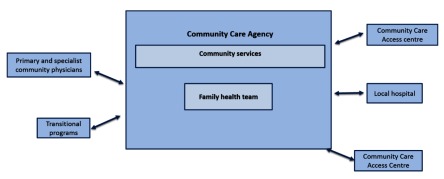
Community care agency.

The first case is a not-for-profit community care agency located in Toronto. The emphasis for integration at the organizational level for this case has been to expand services offered within the organization, while seeking some strategic partnerships with three main external healthcare organizations (see Figure 4).

The agency includes two organizational arms: community services and a family health team (with a main site in the North and a satellite site in the east end of the city). The community services branch of the organization was established in 1976 with the aim of delivering community care services to the Chinese immigrant population in Toronto, beginning with meal delivery. Over the years, services expanded to include a wide array of community support services such as adult day programs, transportation, meals on wheels, friendly visiting, homemaking, chronic disease management programs, home care services, assistive and supportive living services, community programs, and social and wellness programs. To meet the needs of their clients for integrated community and primary care services, in 2007, they established two family health teams within the organization. Family health teams are inter-professional teams of providers delivering primary healthcare services [25]. They also expanded their clientele base, now delivering services to clients from a variety of ethnic and cultural backgrounds, speaking many different languages. A foundation was created in 2006 as a registered not-for-profit entity which aims to fund community care services.

The community care agency works with three main organizational partners: primary and specialist community physicians, a local hospital and Community Care Access Centres. Community physicians and specialists who deliver primary and specialty care to clients are often from ethnic and language groups represented by the community care agency’s clientele. The community care agency also has a strong partnership with a local hospital with whom they deliver their two transitional programs; Assess and Restore and Virtual Ward. The partnership involves connecting nurses from the hospital to a team of providers from the community care agency to provide services to help patients transition from hospital to home. The community care agency is also under contract with two Community Care Access Centres to deliver home care services. Clients may also receive home care services from other home care providers (contracted through the Community Care Access Centre) when patients require home care services that are not offered by this agency.

### Professional integration

Most of the professional integration in this case example occurs within the organization with some examples of inter-organizational professional integration as well. The addition of the Family Health Team has supported integration within the organization by: 1) offering clients access to a multi-disciplinary primary care team which includes nurses, social workers, dietitians and pharmacists; and 2) by connecting primary care services at the Family Health Team to home and community services offered through the community services branch by both formal and informal methods. The Family Health Team staff members are able to work together with providers in the community services branch through formal means such as case conferences and also through informal communications to support coordinated care delivery.

Beyond the addition of the Family Health Team, the organization adopted two programs for older adults to support professional integration of primary and community based services: 1) The Geriatric Assessment and Intervention Network (GAIN) clinic, an interdisciplinary team which develops integrated patient care plans with client’s primary care doctors; and 2) the Program of All-Inclusive Care for the Elderly (PACE) model which uses care coordinators to integrate patient services across the organization. The organization also has care coordinators who work in the Family Health Team, and as part of other programs (such as assistive living and the adult day program). Providers within the community services arm of the organization have a single electronic information system to easily share patient information and communicate. The Family Health Team has a separate electronic medical record, however all providers within the Family Health Team can see patient information within the record.

The Virtual Ward program with the hospital (described above) and connection with Community Care Access Centres offers professional integration to clients as well. Professional integration through the Virtual Ward is supported by having providers from the hospital and agency working together in a single team to support care transitions. Professional integration with the Community Care Access Centres is mainly supported through ongoing communication about client needs between care coordinators at each agency.

## Case – Integrated Client Care Program

### Organizational integration

**Figure 5:**
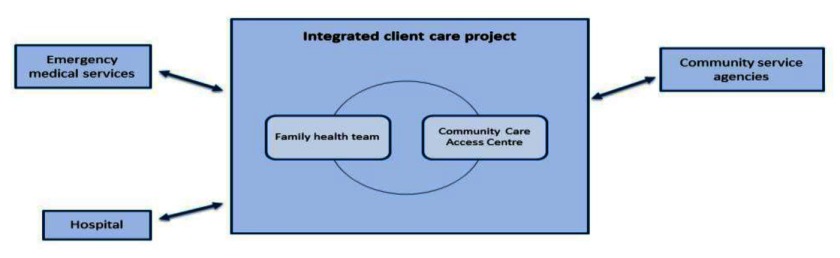
Integrated Client Care Program.

This second case has a much stronger emphasis on inter-organizational integration as compared to the community service agency described above, and focuses on a particular model of care, the Integrated Client Care Program (ICCP) rather than an organization. The Integrated Client Care Program is a model of integrated care based on a partnership between one Family Health Team and one Community Care Access Centre in downtown Toronto. The ICCP is focused on the top 1–5% of frail older adults in need of integrated care services. Clients are assessed using a standard clinical assessment tool, the RAI [27], and if deemed eligible they can access (capitated) services without any cost to them. Figure 5 visually depicts organizational integration in the second case.

Organizational integration is supported primarily by embedding a Community Care Access Centre care coordinator directly into the Family Health Team. Patients are able to access inter-disciplinary primary care through the Family Health Team (as is the case with other Family Health Teams like those described above). In addition, patients can access home and community care services through Community Care Access Centres care coordinators who develop care plans for patients and coordinate delivery of home and community care services by other agencies under a contract model [28].

In addition to the Family Health Team and Community Care Access Centre there is a partnership with Toronto Emergency Medical Services (providing ambulance and emergency services) so that providers at the Family Health Team and care coordinators can be made aware when clients access emergency services.

The Family Health Team also has a number of formal and informal partnerships outside of the ICCP. These include partnerships with: 1), other community service agencies who can provide community support services not offered by Community Care Access Centres (such as homemaking, friendly visiting, meal preparation and transportation); 2) the local hospital (initiated through a Virtual Ward program which supports transitions for clients going from hospital to home); and 3) specialists via a telehealth service (telemedicine IMPACT plus program).

### Professional integration

Patients enrolled in ICCP are able to access multi-disciplinary primary care services through the Family Health Team as well as home and community care services offered by the Community Care Access Centre and other community support agencies. These services are coordinated by the care coordinator embedded within the Family Health Team. Professional integration is supported through the process of delivering care to ICCP patients in which Family Health Team providers work closely with the care coordinators to determine what services are required. Patient visits in the clinic or in the home may include different providers from the Family Health Team such as physicians, physician assistants, social workers, pharmacists, or any other provider at the team along with care coordinators who can then coordinate care needs at a team meeting with the patient. Providers are encouraged to work to their full scope of practice as part of the team, and providers will confer and refer to each other based on their different areas of expertise.

Professional integration is also supported through information sharing which occurs both formally and informally. Informal discussions and case conferences are used by the ICCP team to coordinate care delivery to patients. Information sharing is also enabled by technology. Having the case coordinator embedded in the Family Health Team allows the coordinator to have access to the patient information housed at both the Family Health Team and Community Care Access Centre which supports the ability to integrated care delivery across the team. There is also an integrated system between the Family Health Team, hospital and Toronto Emergency Services so the entire care team can be kept up to date on what is happening with ICCP patients.

The Family Health Team offers other services that serve to integrate community and primary care services which ICCP clients are able to access. Programs described above like the Virtual Ward and IMPACT plus rely on multi-disciplinary teams to deliver care to clients. The social worker in the Family Health Team plays an important role in connecting ICCP and other clients to community service agencies. Providers at the Family Health Team refer clients to the social worker to make these connections and follow-up on patient care received outside of the Family Health Team.

## Case – Community Health Centre

### Organizational integration

The third Ontario case is a Community Health Centre which provides services for patients in a large area in the west end of Toronto and includes four sites: two are “hub” sites which offer a full range of services, and the remaining two are community sites which offer more limited services. The Community Health Centre offers primary care services, counseling, case management, chronic disease management programs, dietetics, foot care, care for older adults, maternal and new baby programs, community support programs, and outreach and health promotion services. The Community Health Centre tends to serve new immigrants and disadvantaged populations in the catchment area.

**Figure 6:**
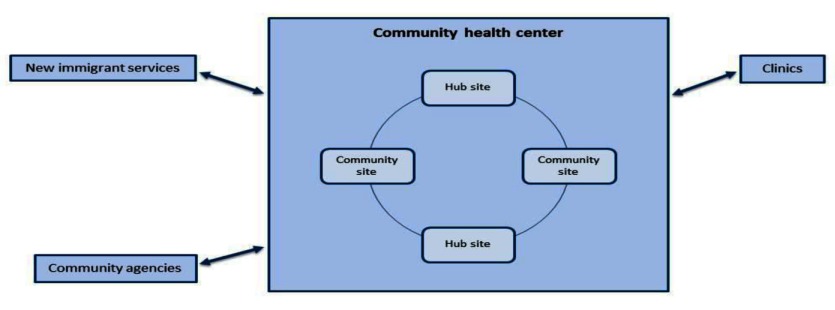
Community Health Centre.

Organizational integration between the Community Health Centre and services including community support services, new immigrant services and clinics such as disease management program is supported through co-location and informal and formal connections (as illustrated in Figure 6). The hub sites at the Community Health Centre are further co-located with other community services including counseling, employment, legal, dental as well as older adult and youth groups. The hubs also include community kitchens and spaces that can be used by other local community groups. The Community Health Centre also works with local community agencies informally to connect patients to needed services. Many of these connections are based on personal relationships between providers at the Community Health Centre and other agencies.

### Professional integration

Patient care is coordinated through case managers and social workers who advocate on behalf of patients, provide counseling and crisis intervention, and provide information and referrals to services within the Community Health Centre and with other partners to meet the health and social needs of patients. Case managers and social workers will also talk with primary care providers at the Community Health Centre about patient needs to trouble shoot issues through formal monthly meetings, via messaging in the electronic medical record, or informally in passing conversation. In some instances, patients may sit down with multiple providers from the Community Health Centre to work out a care plan. Any individual with or without identification or a health card can access all services at the Community Health Centre. All services are free and interpreting services are offered if needed.

Hub sites offer a particularly important means of professional integration as primary care providers from the Community Health Centre can physically walk over to community agencies housed in the same building to connect patients to services and follow-up with other providers. At one of the hub sites there is a shared staff lounge where staff from all agencies can take breaks or eat lunch together and socialize, offering informal opportunities to connect about patients.

## Three cases studies in New Zealand

### New Zealand system context

The New Zealand health system is a mix of public and private ownership across a wide range of health services which have been repeatedly restructured over the last three decades. The health system is funded mainly from general taxation (9.5% GDP) with 20 district health boards (DHBs) receiving three-quarters of this funding to plan, purchase and provide health services for their geographic populations. This includes funding primary care, hospital services, public health services, aged care services, and services provided by non-government health providers including Māori and Pacific providers. Primary care is mostly provided by general medical practices run as state-subsidised small businesses clustered into non-profit Primary Health Organisations (PHOs). Every person in the country is expected to register with a PHO, which receives capitation funding on their behalf through the DHBs. Māori and Pacific providers can form PHOs or can contract separately with government agencies. Accident services are funded by the Accident Compensation Corporation.

The foundational New Zealand Health Strategy [29] signaled the development of community-based primary healthcare. When it was recently “refreshed” for the next 10 years [30], the first acknowledged guiding principle was the special relationship between Māori and the Crown under the Treaty of Waitangi, signed in 1840. The Treaty guarantees partnership, participation and protection for Māori and the Government recognizes this as an obligation to ensure that Māori have at least the same level of health as non-Māori. The current status, however, is that Māori, Pacific peoples and those with lower socioeconomic status experience much higher levels of chronic disease, earlier in life [31], resulting in higher morbidity and lower life expectancy [32].

Whānau Ora (family health) is a national multiagency approach to reduce inequalities and improve the health and wellbeing of Māori, including older adults with chronic illnesses. The Government has invested NZ$164 million and NZ$40 million was released in the May 2016 to implement this policy by empowering whānau through community-based primary healthcare over short, medium and longer-terms. Key outcomes, framed around empowering whānau into the future, are that whānau be: self-managing and empowering leaders; leading healthy lifestyles; participating fully in society; participating confidently in Te Ao Māori (the Māori world); economically secure and successfully involved in wealth creation; cohesive, resilient and nurturing; responsible stewards of their living and natural environments. Clearly these outcomes are aligned with integration of health and social services that goes beyond the traditional siloed approaches.

The New Zealand Disability Strategy [33], The Positive Ageing Strategy [33], and The Health of Older Persons Strategy [34] focus on older adults and those with long-term conditions recognising the need for improved co-ordination of community-based primary healthcare around the needs of older adults and a greater emphasis on health promotion and disease prevention to assist with positively ageing. Implementation action is a specific priority of the Ministry of Health.

## Case – Ki A Ora Ngatiwai

### Organisational integration

Ki A Ora Ngatiwai Trust was established in 1999 by Ngatiwai, a Māori sub-tribe in the North of New Zealand, to deliver primary healthcare and public health services to their community. The Trust serves parts of a regional city and an extensive rural area that includes about 20 000 people of whom about 25% are Māori. Most of the people who use their services are Māori, but not all are Ngatiwai.

Ki A Ora Ngatiwai Trust funding comes from multiple sources which reflect their broad health and social service remit. Sources include the Ministry of Health, Te Puni Kokiri (The Ministry of Māori Development), Northland District Health Board, Te Tai Tokerau Whānau Ora Collective and the two regional PHOs. The Ki A Ora Ngatiwai Trust is not a PHO and, as such, cannot access some funding streams, including capitation for enrolled patients. For this reason they encourage all patients to formally enroll with a PHO for reduced cost access to a mainstream general practice, and do not have a registered population who exclusively use their services. Some patients do use only Ki A Ora Ngatiwai Trust, but many also use mainstream general practice services. In the context of this study focusing on older persons, it is notable that Ki A Ora Ngatiwai Trust explicitly reflects traditional Māori valuing of older adults (kaumatua and kuia) and therefore their health.

The Trust has formal contracts to deliver primary healthcare services, whānau ora (family/whānau health navigators), kaumatua/kuia (older adult) services, mobile nursing services, care coordination, cervical screening and immunisations, health promotion, rheumatic fever outreach to schools and families, youth health (including mental health) which includes school outreach, and alternative education services. They conduct more than 6500 patient consultations per year. Primary healthcare is delivered in a central suburban clinic, at three rural clinics or in patients’ homes. The public health and social services are delivered at a wider variety of venues including schools and marae (traditional meeting places). The workforce comprises approximately three part-time general medical practitioners, one nurse practitioner, four nurses, twelve community health workers and visiting allied health. Their work thus overlaps considerably with, but extends well beyond the services offered by traditional general practice.

**Figure 7:**
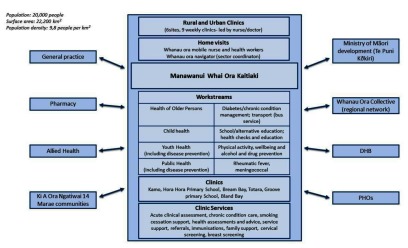
Ki A Ora Ngatiwai.

Ki A Ora Ngatiwai Trust provides Whānau Ora services under contract, although the whole organisation has been imbued with the underlying philosophy long before it became national policy. Rather than multiple providers from different sectors being involved with a single whānau the goal is to offer a simplified service coordinated by a navigator. The navigator supports whānau by linking them with government agencies or specialist services that can progress solutions they have identified in partnership (See Figure 7).

### Professional integration

Apart from formal agreements, Ki A Ora Ngatiwai Trust has a long list of relationships with health and social agencies based on long-standing inter-personal relationships and shared values, which place Ki A Ora Ngatiwai in a strong position to access a wide range of health and social services for their patients. On the basis of these relationships further services are delivered. For example, local general practices will ask them to contact and manage patients whom the practice has been unable to contact. Similarly, the local hospital regularly notifies them when a known Ngatiwai patient is discharged from hospital, and Ki A Ora Ngatiwai staff will contact that patient and follow them us as needed.

## Case – Manawanui Whai Ora Kaitiaki programme within Hauraki Primary Health Organisation

### Organisational integration

Manawanui Whai Ora Kaitiaki (MWOK) is a long-term condition management programme for patients from six general practices covering about 12,000 patients in a geographic area that includes mostly rural dwellers, rural service towns and parts of one regional city. This population has a high proportion of Māori (34%) and people in the lowest socio-economic quintile (36%). The population is older than the national average.

Manawanui Whai Ora Kaitiaki grew directly from a pilot run by Healthcare of New Zealand, a private provider of national services to older persons, serving different general practices in the same geographic area. The pilot came to an end after a year and Healthcare of New Zealand initially formed a partnership with Hauraki Primary Health Organization which soon took over the whole running of the programme. In the pilot the programme ran largely independently of general practice, while linking information back to the practices. Manawanui Whai Ora Kaitiaki operates as an extension of six general practices which are part of the Hauraki Primary Health Organization which includes 32 general practices with about 112,000 patients enrolled at the end of 2015.

**Figure 8:**
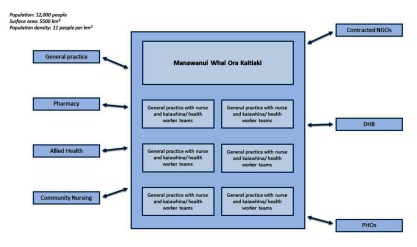
Manawanui Whai Ora Kaitiaki programme.

The service is funded in part by redirecting funds from a national long-term condition programme (CarePlus) which targets support to about 5% of the population mostly through free visits to nurses in general practice. Manawanui Whai Ora Kaitiaki was expected to target about 5% of the population but so far serves nearer to 0.5% at any one time. Neither CarePlus nor Manawanui Whai Ora Kaitiaki target the older population exclusively but this is their main clientele (See Figure 8).

### Professional integration

Manawanui Whai Ora Kaitiaki consists of six teams of a nurse and a kaiawhina (a community health worker) with one team in each of the six practices participating to the programme. The service is designed to be culturally sensitive to Māori patients but serves patients of all ethnicities. It is designed to improve access to services, mainly by visiting patients at home, and to increase intensity and coordination of services. It has a specific brief to attend to both health and social issues. Patients continue to receive general practice care and are typically discharged from the service after about six months. The medical record stays with the general practice and Manawanui Whai Ora Kaitiaki staff come to the practice to view or add to the medical record.

The partners are those of traditional general practice in this region and those contacted directly by the Manawanui Whai Ora Kaitiaki teams. Prescriptions need to come from a general practitioner or hospital doctor, but otherwise, the general practice and Manawanui Whai Ora Kaitiaki teams can interact with and refer to the same agencies. These include hospital inpatient services, hospital outpatient medical and allied health services, homecare support and government and non-governmental social agencies.

## Case – Canterbury Clinical Network

### Organisational integration

A key focus of Canterbury DHB, over the past 15 years has been to improve integration of primary, secondary and tertiary health services within a wider context of quality system improvement. The DHB has some 130 general practices, 115 community pharmacies, 110 dentists, approximately 100 aged care facilities (residential care homes) and more than 50 mental health providers [35]. The quality improvement process to improve integration commenced in 2006–2007 with the identification of new “health pathways” across general practice, social care, and hospitals [36, 37]. Activity-based payments for hospitals were replaced with bottom-up budgeting for each specialty, and contracts for externally provided services were moved from a competitive, often fee-for-service basis to a form of alliance contracting where maximum collective gain can only be realised if all parties support one another and agree to share any losses [38, 39]. We note that, although the changes were started in 2006–7, they were greatly accelerated following major earthquakes in Christchurch in late 2010 and early 2011, in which there was great loss of life and infrastructure.

The programme of systemic change within the DHB has been driven by the Canterbury Clinical Network. The Network includes urban and rural general practices, practice nurses, pharmacists, contracted service providers (including home care), allied health professionals, community nurses, the Canterbury DHB, Primary Health Organisations and general practitioner groups. The Network comprises the Alliance Leadership Team, Alliance Support Team, workstreams and service level alliances and workgroups. The Alliance Leadership Team is made up of clinical leaders, key health managers and other experts from across the Canterbury health system. It operates to ensure a high degree of collaboration, engagement and innovative thinking around transforming services and work practices and is supported by the Alliance Support Team. The health of older adults, mental health, rural health and child and youth workstreams propose transformational service improvement and identify areas requiring redesign and innovation. The service level alliances comprise a number of identified areas, including urgent care, community services, pharmacy, radiology and rural funding. Their role is to build on the guidance developed by the workstreams and design and plan the delivery of a service or group of services in a specific area of health and social services within a defined scope.

**Figure 9:**
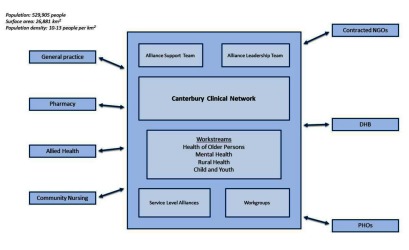
Canterbury Clinical Network.

The wide scope of the evolution of services within Canterbury DHB has necessitated the inclusion of a large number of key organizational partners within Canterbury Clinical Network. The partners include the DHB in its role in planning, providing or purchasing the best possible health and disability services for the diverse and changing Canterbury population. Primary care has representatives from the three Primary Health Organizations funded by Canterbury DHB to ensure the provision of essential primary healthcare services, mostly through general practices, to those people who are enrolled with the Primary Health Organisation. In addition non-government organizations contracted to provide disability support within the home, private contracted laboratory, community pharmacy and radiology services and St John, a charitable organisation that delivers ambulance, community and health services (See Figure 9).

### Professional integration

Key services that have developed by Canterbury Clinical Network to support older people within community-based primary healthcare are: the Acute Demand Medical Service; the Community Rehabilitation, Enablement and Support Team; the Medication Management Service and Pharmacy Service Level Alliance; and Care Coordination.

The Acute Demand Medical Service is aimed at preventing hospital admission and provides both a means for general practice to give patients support so that they do not need to go to hospital, and a means for the hospital to discharge patients from the emergency department, or from medical and surgical admission wards, without the need for a hospital stay. For any patient who would otherwise have been sent to the emergency department or admitted to hospital, general practice teams can access DHB funding and services, in order to prevent hospital use.

The Community Rehabilitation, Enablement and Support Team is a community based service and aims to reduce inpatient length of stay and readmission to hospital and delay entry into aged residential care for older people and those with complex long-term conditions. Case managers seek to ‘pull’ patients out of hospital, with clients receiving varying levels of support of up to four visits a day, seven days a week at the most intensive. Goals are agreed with patients and, depending on severity, the service lasts from two to six weeks, though typically four to six weeks. The model focuses on rehabilitation, the support being not just medical or nursing but assisting patients in being able to shop again, reconnect with friends and rebuild social networks.

The Medication Management Service and Pharmacy Service Level Alliance initiatives involve pharmacists and general practitioners working together flexibly to review the medication of patients taking multiple therapies in their own homes. The aim is to reduce medication-induced admission to hospital or rest homes.

For older adults and those with long-term conditions, the coordination of care within the Canterbury system occurs in partnership between primary care and Older Person's Health Specialist Service, which comprises a number of services, including two interdisciplinary Community Services Teams that serve defined geographical areas of the region. A case manager, linked to the referring general practice, is identified for each referral.

## Discussion

The cases presented in this paper constitute exemplar models of integrated care implemented in three jurisdictions. Using the Rainbow Model of Integrated Care as a framework, we described the “meso” level, organizational and professional integration of each case. This framework allows the use of similar concepts in the description of the cases, which facilitates a preliminary exploration of the similarities and differences between these models of care and can help point to new areas of exploration.

Although each case aims to improve integration of community-based primary healthcare services for older adults with multiple care needs, these models are structurally different. The Quebec cases portray local health networks that strongly encourage organizational integration with professional integration being facilitated through co-location and standardized assessments. In Quebec, the three cases have the same structural model of integrated care due to a system context which favored a top-down approach of implementation at a very large scale. Conversely, the variability between cases showed that an autonomous space in the manner to reach targets defined by the central level of governance.

Ontario, on the other hand, had a system context which encouraged diversity, leading to three fairly different approaches to integrated care that range from emphases on intra to inter organizational collaboration and coordination. In general, the emphasis in Ontario is on supporting professional integration to bring more services and providers under one organizational umbrella (as is the case with the community agency) or through co-location (as is the case with the Community Health Centre). While the Integrated Client Care Program does demonstrate a focus in inter-organizational linkage this is a much smaller example that those from Quebec with a focus on a small population of high-needs complex older adults.

In New Zealand, cases present different vision of network scopes, inclusion of a varying number of key social and health services agencies, different target populations, and varied funding sources. The New Zealand government has established policy objectives that span health and social ministries and allow for diversity of service delivery. But one aspect is very original and important to understand from these innovations: a much greater emphasis on ethnic population, values, beliefs, needs and, especially, the particular way to adjust the type of response is a relevant component of the integration vision. The health needs of indigenous Māori are acknowledged as a central tenant of national policy. In all three cases funding has been specifically realigned from multiple sources (Ki A Ora Ngatiwai and Canterbury Clinical Network) or re-directed from an existing long term condition programme (Manawanui Whai Ora Kaitiaki) to enable organisational integration. This has led to professional integration, mostly by nurses and unregistered health workers, seeking to improve access ‘to and through’ primary health care and hospital services. Home-visiting is a feature of all cases and a Maori cultural approach is central in two cases.

Another important aspect is some cases focused not only on older adults with multiple care needs. A prime example is the Community Health Centre in Ontario who have a mission to support broad community and population health and therefore aim to support anyone experiencing multiple care needs rather than just focusing on older adults. This also points to the fact that complex care needs can be experienced across the lifespan [40]. Integration for some subpopulation group may leads to more fragmentation into more subpopulation groups.

An important mechanism of coordination implemented in all the nine cases studied is a person in charge of the coordination of services for patients with multiple needs. These dedicated coordinators were alternatively described as case manager, care coordinator or navigator. An interesting observation across the cases is the need to identify a professional in charge of the coordination/navigation when the patient requires services from several providers and organizations. Regardless of the context of implementation and the population target, this mechanism seems to be a relevant component for the coordination of patients with multiples needs.

Future work should explore the other parts of the Rainbow model not explored here. In particular a deeper look at how these differences in organizational and professional integration impact on patient’s experience of integration can help to elaborate on the micro level, or clinical integration. Given the importance of both formal and informal relationships in these studied cases, it would also be important to dig into the normative processes that support integration across macro to micro levels. How relationships, culture and norms influences these models will be keys to unlock how such different models have been successfully implemented.

## Conclusion

This paper present an overview of nine examples of integrated services for older adults in three jurisdictions. The examples serve as valuable case studies because they represent different contexts and implementation of care. The “meso” level of integration based on the Rainbow Model of Integrated Care framework is used to describe and classify organizational and professional components of integration across the nine case studies. Despite many differences between the cases in terms of system context, duration of experience, scope, target population, resources within and across organisations, it appears that some components are transversely present. For example, development of partnerships, work in inter-professional teams, common use of clinical tools, electronic information systems and dedicated coordinators are core components of the cases. In all cases studied, the amount of available services is large and diversified and seems in line with the overall needs of the population, but specific place and role of family doctors in the implementation of these different models of integration is not clear. However, the number of organizations and providers involved makes the systems of care complex and therefore requires support for users and caregivers for navigation and coordination. In each case example, the design of organisation and professional integration acts as a mediator between policy and clinical delivery. Studying the implementation of integrated care in each case offers an important opportunity to better different approaches to achieve integrated care.
